# How best to handle the evidence in a cost-effectiveness analysis of TAVI in the UK

**DOI:** 10.1186/1745-6215-12-S1-A46

**Published:** 2011-12-13

**Authors:** Aileen Murphy, Elizabeth Fenwick, Andrew Briggs, Matthew Neilson

**Affiliations:** 1 Department of Economics, University College Cork, Ireland; 2 HEHTA, University of Glasgow, Glasgow, Scotland

## Aims

Despite available literature and results from RCT the question of whether Transcatheter Aortic Valve Replacement (TAVI) is cost-effective, and for whom, persists in the UK. This paper investigates how evidence from published literature, RTC and registry data can be employed and best synthesised to determine the cost effectiveness of TAVI and establish for whom is TAVI most suitable.

## Methods

A decision analytical model, incorporating a short–term decision tree and long-term Markov model were constructed. The use of Monte Carlo simulation and Value of Information analysis enables both the cost-effectiveness of TAVI to be estimated, based on the existing information, and the value of, and requirements for, further evidence collection to be determined. The model allows for the heterogeneous patient population by considering different three patient risk cohorts (low, medium and high). Initially the model was populated using the best available literature; then updated when RCT results became available. However, the trial data did not fully reflect UK practice as such the model was updated with UK Registry data. Probabilistic analysis is undertaken at each stage and results are presented for the cost-effectiveness and the value of undertaking further research.

## Results

The initial model results revealed that TAVI is cost-effective for inoperable patients only compared to medical-management with an ICER of £22,603. The EVPI per high risk patient ranges from £462 to £1,277. When the results from the PARTNER trial were incorporated into the model, TAVI remained cost-effective for these patients compared to medical-management, ICER £25,875. The EVPI using the PARTNER evidence ranged from £69 to £1,170 per patient. At each of these stages, there is potential value in undertaking further research in this patient sub-group within the UK.

## Conclusions

This paper reveals that a flexible model accommodating an evolving evidence base is a necessity when dealing with novel technologies for which data is scarce, such as TAVI. While the US RCT evolves the database, the suitability of this data is questionable for a UK cost-effectiveness model. Similarly the suitability of the UK TAVI registry is also undefined as it is not randomised. This paper examines how best to synthesise and incorporate these data into a decision analytical model to reveal for whom TAVI is cost-effective in the UK.

